# Impact of consumer power on consumers’ reactions to corporate transgression

**DOI:** 10.1371/journal.pone.0196819

**Published:** 2018-05-03

**Authors:** Takaaki Hashimoto, Kaori Karasawa

**Affiliations:** Department of Social Psychology, Graduate School of Humanities and Sociology, The University of Tokyo, Bunkyo-ku, Tokyo, Japan; National Taiwan University, TAIWAN

## Abstract

We addressed how individuals’ power influences their judgments regarding corporate transgressions. Based on the Situated Focus Theory of Power, which theorizes that powerful people respond more in accordance to circumstantial factors, we tested the interaction of power and the type of corporate discourse offered by the accused company. Across two studies (overall *N* = 216), we experimentally primed power (Study 1) and manipulated participants’ sense of direct control over the company (Study 2). We consistently found an interaction effect of power and corporate discourse on people’s negative attitudes toward the company—particularly on the unwillingness to use the company’s products. Particularly, high-power individuals were prone to strongly vary their attitudes based on the mitigative/non-mitigative nature of the discourse, while those low in power were unsusceptible to the type of discourse. The results suggest how the potential rise of consumer power in society may critically influence the consumer-corporate relationships following corporate transgressions.

## Introduction

Along with the society’s heightened emphasis on issues of corporate social responsibility, research has increasingly highlighted how consumers view and respond to corporate misconducts. The factors include the events’ situational characteristics—such as the severity of harm-giving and how a blameworthy firm reacts to crises [1, 2]—and the consumers’ personality traits [1, 3]. Consequently, consumers experience negative emotions [3, 4]; generate impressions on the overall unethicality of the company and its brand [5]; and potentially exhibit negative behavioral responses such as avoiding the company’s brand, spreading negative word of mouth, or protesting [4].

Our study adds to this line of research by considering the variable of *power*. Power is defined as a person’s capacity to modify others’ states and control situational outcomes [6, 7], such as by providing or withholding material or social resources or by administering sanctions. Studies in the domain of consumer psychology have addressed the effects of power on people’s consumption tendencies, demonstrating how powerless people prefer status-related goods [8], whereas powerful people value the utility of the product [9] (see also [10]). The current study incorporates a different angle and considers how power shapes the relationship between a consumer and a company.

Generally, consumers are unlikely to feel that they possess the power to directly control or influence companies’ outcomes [11]. Such views have appeared in past discussions on how consumers react to corporate transgressions. For instance, when consumers feel a need to punish an irresponsible company, the power to exert influence over an entire company is out of the hands of ordinary individuals [12]. The low entitative nature of companies also contribute to the general sense of difficulty in sanctioning the company, as people see no “bodies to kick” nor “souls to damn” [13]. Because they are incapable of direct retaliation against the company, consumers take more indirect and covert means of negative responding such as grudge-holding and withdrawal [14], and blame its human representatives rather than the company as a whole [15, 16].

Meanwhile, another line of discussion suggests that such power structure is now changing. That is, the consumers may be gaining power since the Internet has introduced people with means to voice and more directly influence companies and their brands [11, 17, 18].

The discussions imply that the psychological states of power—either feeling powerless or powerful—is a crucial component of consumer–corporate relationships. However, to date, there is a dearth of research that empirically tests the impact of power on consumer behavior, let alone in a corporate transgression context (e.g., [14]). In the current research, we induced people’s subjective experiences of power and investigated its effects on their judgments about cases of corporate misconducts.

As a theoretical framework to understand power’s effects, we adopted the Situated Focus Theory of power [19, 20]. The framework, as we detail in the next section, suggests that when people subjectively feel powerful, they behave in greater concordance to the situationally-activated goal. We predicted that when people encounter a corporate transgression and are offended by such information, those experiencing high power would respond with greater negativity toward the responsible company. At the same time, we predicted that powerful people would show a greater degree of conciliation if situational factors trigger responses in such direction (e.g., the company offers an acceptable apology).

### The Situated Focus Theory of Power

The Situated Focus Theory of Power proposes that power influences people’s basic social cognition and behavioral tendencies. It claims that “the responses of powerful individuals reflect more unequivocally the influences (e.g., needs, desires, goals, priming, affordances) that operates on a moment-to-moment basis” ([20], p.143). When situational proponents activate a goal at a given moment, powerful individuals show higher accessibility to that goal [21], and are able to execute in an approach-oriented, disinhibited manner the actions driven by that focal goal [6, 22–25]. As a result, behaviors of powerful individuals vary greatly according to the call of the situations. In support of the theorization, studies report that powerful people may behave more prosocially or more antisocially than powerless people depending on the task demand [23], or that they react consistently with the affordance of the given situation [26].

In light of the Situated Focus Theory, the current research examined the effects of power on consumer behavior, taking into account the potential interaction with a contextual factor. Namely, we manipulated the type of public discourse delivered by the company following its transgression. While a company’s post-crisis discourse may generally involve many elements (e.g., denial or admittance of responsibility, confession, apology [27]), we compared the effects of a discourse which does or does not include *mitigative* factors [28]—that is, admittance of responsibility and apology.

When the company offers a neutral discourse which lacks any mitigative elements, people’s focus will be on the transgression itself. In such case, people’s reactions would be predominantly negative, involving intentions to penalize and/or avoid the company [4, 12, 14, 29]. Given that power contributes to the facilitation of situationally dominant responses, we predicted that people’s punitive tendency would be strengthened by a heightened sense of power. In support of such an assumption, studies on interpersonal transgressions have shown that people in a powerful mind-state indicate more explicit punitive judgments toward moral violators [30, 31].

Meanwhile, an apologetic discourse, when given appropriately, would trigger people’s conciliatory responses. Studies demonstrate that apologies given at the organizational level foster people’s satisfaction [32] and recovery of the corporate image [33]. More broadly, a great number of interpersonal-level research highlights how an apology triggers a variety of psychological reactions within its recipients, including empathy [34, 35], positive impression about the target [36, 37], perceptions about expectancies of future transgressions [38], and ultimately forgiveness toward the target [39–41]. Forgiveness, specifically, is a process requiring cognitive control, in that one has to inhibit the preexisting negative tendencies and coordinate responses to the now-salient goal of reconciliation [42, 43]. Power, on the other hand, has been found to associate with cognitive control [44], and the Situated Focus Theory suggests that “powerful people will change more their responses … as a function of changes in the situation” ([19], p. 258). Based on such lines of discussions, we predicted that when people perceive mitigative elements in a company’s discourse, those experiencing high power would be more strongly inhibit their negative responses and react more benevolently than those without power.

### Overview of the studies

In sum, we conducted two studies where we manipulated people’s sense of power and subsequently presented them with cases of corporate misconduct. In the corporate discourse they observed, we operationalized whether they perceive mitigative elements (admittance of responsibility, apology). We predicted that in the absence of such elements, for instance when the company is yet to make any public remarks, a subjective experience of high power would enhance people’s initial and dominant reaction to respond negatively toward the company. Meanwhile, in the presence of a mitigative discourse, powerful individuals would respond with less negativity toward the company than their low-power counterparts.

A major distinction between the two studies was how we operationalized power. In Study 1, we primed participants with sense of power via a role-assignment paradigm, and presented them with the corporate scenario as part of a seemingly unrelated task. The paradigm allowed us to test the effect of power as a psychological state activated independently from the focal task in question [23]. In Study 2, we manipulated power by inducing participants to feel that they have actual influence over the accused company. To create a condition where participants realistically experience such form of power, we gave an impression that they can influence the company through responding to the given survey. By implementing two variations of manipulation and testing for consistency in their effects, we aimed to capture the general effects of the subjective experience of power on consumer responses.

The present study considered several dependent variables. In Study 1, we focused on a variable directly related to consumption behavior: people’s reluctance to use the company’s products. In Study 2, we examined a wider range of dependent variables in addition to people’s reluctance. Along with buying and using of products, a core element of consumer-corporate relationship is how consumers regard the company as a socially responsible agent in society [45]. Studies show that, in face of corporate misconducts, factors such as moral emotions [3, 4], punitive intentions [12], trust [46], and forgiveness [2] underlie consumer responses. We included such variables in Study 2 and tested whether power has a broad effect on them.

## Ethical concern

The two studies reported in this paper both received prior approval from the Ethics Review Board of the Department of Social Psychology of The University of Tokyo. We informed all participants of their participation being completely based on free will, that they are free to withdraw from participation at any time, and that the data will be processed anonymously. In Study 1, we obtained written informed consent; in Study 2, we regarded a completed questionnaire as a sign of consent.

## Study 1

### Material and methods

#### Participants

We recruited participants via a sample pool available at our department and also through notices posted around the campus. Without setting the sample size a priori, we collected data for a predesignated period of four weeks. As a result, sixty-seven Japanese undergraduate students (48 men, 19 women; mean age of 20.9, *SD* = 1.28) showed up to participate in the experiment. We performed the analyses based on this dataset and did not collect additional data after the analyses.

We ran the experiment with two participants per session, and in nine of the sessions where a second participant did not show up, a confederate substituted for the role. We assigned each participant to one of four conditions in a 2 (low vs. high power) ✕ 2 (mitigative vs. neutral discourse) design.

#### Power manipulation

We adopted the paradigm from Guinote [47], where the experimenter randomly assigned participants into the role of either the high-power Judge or the low-power Worker. The experimenter told participants that the Worker will perform tasks under the instructions given by the Judge, and the Judge will then evaluate the Worker’s performance. Furthermore, the Judge’s evaluation will determine the amount of the Worker’s reward, varying from 600 to 1000 Japanese yen (JPY), whereas the Judge will receive a fixed reward of 800 JPY. Upon receiving the instructions, the participants responded to a set of manipulation check items on their experiences of power (i.e., six items such as “I can exert influence over my partner during the task” and “The amount of payment I receive depends on my partner’s evaluation”) on a scale from 1 (*strongly disagree*) to 7 (*strongly agree*).

#### Corporate transgression

While the experimenter prepared for the pair-task, participants took seats in separated booths and responded to an ostensibly unrelated survey on a computer screen. A fabricated news article described a case where a malfunction of a motortruck—manufactured by a company named Y Motors—resulted in the driver’s injury. In the neutral-discourse condition, the company’s representative mentioned that they were still in the process of investigation and withheld any specific announcement. In the mitigative-discourse condition, the company publicly confessed that they had prior knowledge about the potential deficiency that led to the accident, that they have failed to take full measures in announcing and dealing with the deficiency, and offered an apology (see S1 Appendix for the full vignettes).

For the dependent measure, two items assessed participants’ reluctance to use the target company’s product: “I prefer not to use Y Motors' products” and “If necessary, I am willing to use Y Motors’ products (reverse-coding item).” Participants responded on a scale from 1 (*strongly disagree*) to 7 (*strongly agree*).

Finally, the participants were told that the preannounced pair work will not take place, were fully debriefed, and received a gift certificate worth 1,000 JPY.

### Results and discussion

#### Tests of nonindependence

We ran the experiment in pairs, so we checked the independence of responses from each dyad. For the mean scores of the six items on participants’ subjective ratings of power (α = .86), we found no significant intraclass correlation (ICC < .001, *χ*^2^(37) = 13.74, *p* > .999). Meanwhile, for the two items on reluctance to use the company’s products (*r* = .69), we found a significant ICC of .197 (*χ*^2^(37) = 55.32, *p* = .040). Thus, to account for any nonindependence, we hereafter conducted dyadic data analyses using multilevel modeling [48]. We effect-coded power (low-power = -1, high-power = 1) and corporate discourse (neutral = -1, mitigative = 1), and entered the terms along with their interaction as Level-1 predictors. Being dyadic data, the models also allowed for random effects of the Level-2 intercept. We used HLM software (version 7.03) for the estimations. The results are displayed in Table 1.

**Table 1:** General linear mixed models of dependent variables regressed on power and apology (Study 1).

	Manipulation check on power				Reluctance to use product			
Fixed effects	*b*	*SE*	*t (df)*	*p*	*b*	*SE*	*t (df)*	*p*
Intercept	3.99	0.07	54.70 (37)	< .001	4.80	0.13	35.94 (37)	< .001
Power (P)	1.26	0.08	15.74 (26)	< .001	0.16	0.12	1.41 (26)	.170
Discourse (D)	-0.03	0.08	0.36 (26)	.721	-0.33	0.12	-2.73 (26)	.011
P × D	-0.05	0.08	0.60 (26)	.553	-0.30	0.13	-2.32 (26)	.028
Random effects	Variance		*χ*^2^ (37)	*p*	Variance		*χ*^2^ (37)	*p*
Level 2 intercept	0.00		33.15	.650	0.17		47.15	.123
Level 1 residual	0.41				0.98			

Power was effect coded as: Low-power = -1, High-power = 1. Corporate discourse was effect coded as: neutral = -1, mitigative = 1.

#### Manipulation check on power

With participants’ subjective ratings of power, the mixed model analysis indicated a significant effect of power manipulation, and neither main nor interaction effects of corporate discourse. Participants assigned to the high-power role reported having stronger power over their partner than those assigned with low power (${\hat{y}}_{low-power}$ = 2.73, ${\hat{y}}_{high-power}$ = 5.25).

#### Reluctance to use the company’s products

With participants’ intentions toward the usage of company’s products, the mixed model analysis revealed a main effect of corporate discourse, qualified by an interaction effect of power and discourse. We conducted a follow-up simple slope analysis, first with discourse as the moderator of the effect of power. Under a neutral discourse, the high-power participants expressed stronger reluctance in using the company’s products compared to the low-power participants (*b* = 0.46, *SE* = 0.18, *p* = .016, ${\hat{y}}_{low-power}$ = 4.66, ${\hat{y}}_{high-power}$ = 5.58); whereas, when the company presented a mitigative discourse, the high and the low-power participants did not differ in their unwillingness to use the product (*b* = -0.13, *SE* = 0.19, *p* = .496, ${\hat{y}}_{low-power}$ = 4.60, ${\hat{y}}_{high-power}$ = 4.33). Alternate analysis with power as the moderator showed that the mitigative (relative to neutral) discourse decreased reluctance among people with high power (*b* = -0.63, *SE* = 0.19, *p* = .003, ${\hat{y}}_{neutral}$ = 5.58, ${\hat{y}}_{mitigative}$ = 4.33); whereas, the type of discourse did not significantly alter the response of those with low power (*b* = -0.03, *SE* = 0.18, *p* = .852, ${\hat{y}}_{neutral}$ = 4.66, ${\hat{y}}_{mitigative}$ = 4.60).

Study 1 provided preliminary evidence on how experiences of power influence people’s reactions to a corporate transgression. The high-power individuals indicated a stronger reluctance than to use the company’s products than the low-power individuals—specifically when the firm did not provide a formal explanation and an apology about the event. Furthermore, a mitigative discourse by the company alleviated high-power individuals’ reluctance, while, in clear contrast, the low-power participants exhibited a constant level of reluctance regardless of the type of discourse. In sum, this initial data illustrate how consumers who feel powerful may be more responsive to the type of discourse presented by the company compared to those feeling low in power.

## Study 2

In the second study, we aimed to replicate and extend the findings of Study 1 using a different operationalization of power. To induce participants’ sense of actual power over the target company, we informed participants that their responses to a survey will or will not have actual influence over a case of corporate misconduct. Such form of power—having awareness that one can express and voice one’s opinions—is among the central constituents of people’s general sense of power [49, 50]. Furthermore, voice is one of the strategies that consumers can possibly take in order to exert influence over corporate firms in the actual world (for instance, with the use of the Internet [17, 51]). Thus, we set up an artificial situation and provided participants with an opportunity for such power, and tested its effects based on, not only the reluctance to use the company’s products, but also a wider variety of dependent measures: magnitudes of retributive intentions, negative affect, trust, and forgiveness. Since Study 1 lacked a manipulation check for corporate discourse, we also included items to check how participants perceived the company’s discourse. Also, we checked whether power has any effects on people’s evaluations of the seriousness of the transgression.

### Material and methods

#### Participants and design

We distributed questionnaire packets during two introductory courses (one on psychology, another on linguistics) in a national university in Japan. We obtained responses from a total of 149 undergraduate and graduate students (94 men, 55 women; mean age of 21.3, *SD* = 2.68). The sample size was constrained to the number of attendees of the two courses we had access to. We performed the analyses based on this dataset and did not collect additional data after the analyses.

Each participant received a questionnaire packet on one of four conditions in a 2 (low vs. high power) by 2 (mitigative vs. neutral discourse) design. The front page of the questionnaire ostensibly indicated that the survey was jointly conducted by the Social Psychology Department of The University of Tokyo and the Corporate Ethics Committee, a subgroup of the Japan Joint Association on Industry. We afterwards debriefed participants about this deceptive cover-up story by handing out a 1-page summary of the research objectives.

#### Corporate transgression

Participants read a fabricated online news excerpt depicting a case of a malfunctioning electronic appliance severely injuring its user. As in Study 1, the company (Breton Electronics) offered either a mitigative or a neutral discourse (see S1 Appendix for the vignettes).

#### Power manipulation

Prior to the page on corporate transgression, the questionnaire included a page headed as “Consent on the usage of the survey results.” It described how the collected responses will be used in the project. We described the Corporate Ethics Committee as consisting of various companies and research institutes to deal with issues of corporate ethics and social responsibility. All data obtained through the survey were to be submitted to this committee; however, the subsequent explanations differed between conditions. For the high-power participants, we described that the data would be reported to the business firms involved in the project to be used to improve the firms’ policies and guidelines, thus exerting direct power over the firms’ actions. We provided check boxes for participants to mark if they fully acknowledged that (a) the data would be handed over to the Corporate Ethics Committee and (b) the data would be used to develop ethical guidelines for business firms.

The low-power text described that the data would be reviewed and examined as an internal reference strictly within the committee, and that the findings will be presented only at an academic conference, and thus will not be made open to nor will it influence actual business firms. Participants indicated whether they acknowledged that the (a) data will be handed over to the Corporate Ethics Committee and (b) it will only be used for academic purposes. Nine participants in the high-power condition and five participants in the low-power condition who did not mark both check-boxes were excluded from analyses.

For additional emphasis, solely in the high-power condition, a subsequent page featuring the transgression vignette included a footnote stating that ethical standards to be established based on the current survey will enforce control over the specific company in question.

#### Manipulation check items

A manipulation check item on power asked the following: “I feel that I can voice my views over Breton Electronics.” To measure participants’ subjective evaluation of the company’s discourse, items asked the degree that participants felt “satisfaction,” perceived “sincerity,” or were “unconvinced” (reversed item) by the company’s response (α = .71). Participants responded to the items on scales from 1 (*strongly disagree*) to 7 (*strongly agree*).

#### Dependent measures

Participants responded to all of the items below on scales from 1 (*strongly disagree*) to 7 (*strongly agree*). An item measured the perceived seriousness of the harm caused by the transgression: “The victim (university student) of the incident incurred serious harm.” Two items measured participants’ reluctance to use the company’s product: “I prefer not to use Breton Electronics’ products,” and “I am willing to use Breton Electronics' products if there is such chance (reversed item).” Three items measured participants’ retributive intentions toward the company: “Breton Electronics should be given legal sanctions,” “Breton Electronics should be given societal sanctions,” and “I want Breton Electronics to take responsibility.” Three items measured negative affect toward the company: anger, distaste, and antipathy. We also included a single-item measurement on perceived trust (“I feel trust in Breton Electronics”), and an item on forgiveness (“As a consumer, I forgive Breton Electronics”).

### Results and discussion

#### Manipulation check on power

We conducted a 2 (power) ✕ 2 (discourse) analysis of variance (ANOVA) on the perceived power over Breton Electronics. We found a significant main effect of the power manipulation (*F*(1, 131) = 4.80, *p* = .030, $\eta_{p}^{2}$ = .035), but neither a main effect of discourse (*F*(1, 131) = 0.25, *p* = .619, $\eta_{p}^{2}$ = .002) or an interaction of power and discourse (*F*(1, 131) = 2.35, *p* = .128, $\eta_{p}^{2}$ = .018). High-power participants more strongly expressed that they could voice over the company (*M* = 3.26, *SD* = 1.81) than did the low-power participants (*M* = 2.65, *SD* = 1.32).

#### Manipulation check on corporate discourse

A two-way ANOVA on the subjective evaluation of the discourse revealed a main effect of discourse (*F*(1, 131) = 21.80, *p* < .001, $\eta_{p}^{2}$ = .143), but no main effect of power (*F*(1, 131) = 0.04, *p* = .848, $\eta_{p}^{2}$ < .001) and a power-discourse interaction (*F*(1, 131) = 0.35, *p* = .553, $\eta_{p}^{2}$ = .003). Participants who received the mitigative discourse (*M* = 3.98, *SD* = 1.27) evaluated the company’s discourse as more convincing and satisfactory than those who received the neutral discourse (*M* = 2.98, *SD* = 1.20).

#### Perceived seriousness of the harm

A two-way ANOVA on the perceived seriousness of the harm yielded no significant main effects of power (*F*(1, 131) = 0.01, *p* = .920, $\eta_{p}^{2}$ < .001), discourse (*F*(1, 131) = 1.36, *p* = .246, $\eta_{p}^{2}$ = .010), or their interaction (*F*(1, 131) = 0.23, *p* = .629, $\eta_{p}^{2}$ = .002). Participants generally evaluated the harm as highly serious (*M*_overall_ = 6.25, *SD* = 0.80) regardless of the conditional power and corporate discourse. In such respect (and given that high/low power participants did not differ also in their evaluations of the discourse), we did not find evidence that power had any effect on people’s basic interpretations about the situation.

#### Attitudes toward the company

We first checked the distinctions among the focal dependent variables. With the 10 attitudinal items, we conducted a maximum-likelihood factor analysis with promax rotation. According to a criterion-eigenvalue of 1, we extracted three factors (see S1 Table for the factorial pattern). The three negative affect items (α = .90) and three retribution items (α = .72) each loaded on a separate factor, so we calculated mean scores for each variable to use in the subsequent analyses. The remaining items—forgiveness, trust, and two items on reluctance to use the products—loaded on a single factor. We interpreted this factor as reflecting people’s overall approach/avoidance tendency aimed at the company. However, since existing studies have considered product usage, consumer forgiveness, and trust as distinct constructs [2, 46], and also for the sake of comparability with Study 1, we retained the distinction of reluctance of usage (*r* = .48), trust, and forgiveness to use in the analyses. See Table 2 for the conditional means and standard deviations of each variable, and Table 3 for their intercorrelations.

**Table 2:** Conditional means and standard deviations of the attitude measures (Study 2).

	Low Power		High Power	
	Neutral Discourse	Mitigative Discourse	Neutral Discourse	Mitigative Discourse
	*M (SD)*	*M (SD)*	*M (SD)*	*M (SD)*
Reluctance to use product	4.77 (1.35)	5.17 (1.14)	5.60 (1.22)	4.54 (1.18)
Retribution	4.41 (1.32)	4.81 (0.77)	5.11 (1.02)	4.50 (1.10)
Negative Affect	3.88 (1.56)	4.14 (1.44)	4.54 (1.22)	3.75 (1.22)
Trust	2.56 (1.28)	2.59 (1.37)	2.35 (1.15)	3.26 (1.46)
Forgiveness	3.26 (1.31)	3.28 (1.30)	3.09 (1.29)	3.91 (1.08)

**Table 3:** Intercorrelations of the attitude measures, and the structure coefficients and standardized discriminant function coefficients for the multivariate power ✕ discourse interaction composite (Study 2).

	*r*				Interaction Composite	
	1	2	3	4	*r*_*s*_	Standardized Coefficient
1. Reluctance to use product	---				.90	.81
2. Retribution	.41***	---			.71	.37
3. Negative Affect	.38***	.54***	---		.56	.15
4. Trust	- .56***	- .28**	- .25**	---	- .50	- .04
5. Forgiveness	- .64***	- .27***	- .36***	.58***	- .48	.19

^**^p < .01.

^***^p < .001.

*r*_*s*_ = structure coefficient.

To examine the effects of the conditions on the attitude measures, we ran a 2 (power) ✕ 2 (discourse) multivariate analysis of variance (MANOVA) on the five dependent variables: reluctance toward product usage, retribution, negative affect, trust, and forgiveness. The Box’s *M* test indicated that the data meets the assumption of homogeneity of variance (*M* = 27.22, *p* = .098). The MANOVA results indicated a significant multivariate interaction effect of power and discourse (*Λ* = .90, *F* (5, 126) = 2.89, *p* = .017, *η*^2^ = .103), while neither of the main effects of power and discourse was significant (*Λ* = .96, *p* = .377, *η*^2^ = .041, and *Λ* = .96, *p* = .384, *η*^2^ = .040, respectively).

To understand the multivariate effect, we conducted a post-hoc descriptive discriminant analysis [52, 53]. In Table 3, we indicated each variable’s structure coefficient and standardized function coefficient for the composite variable created for the power ✕ discourse interaction effect. According to the coefficients, the composite dependent variable was primarily composed of reluctance toward product usage (*r*_*s*_^*2*^ = .81) and retribution (*r*_*s*_^*2*^ = .50), while negative affect (*r*_*s*_^*2*^ = .31), trust (*r*_*s*_^*2*^ = .25), and forgiveness (*r*_*s*_^*2*^ = .23) accounted for relatively smaller, yet meaningful, proportions of variance. The standardized coefficients of negative affect, trust, and forgiveness were relatively low, but since they show high correlations with either reluctance toward usage or retribution, it is suggested that their variance in the composite can be credited to factors of reluctance/retribution.

**Fig 1:**
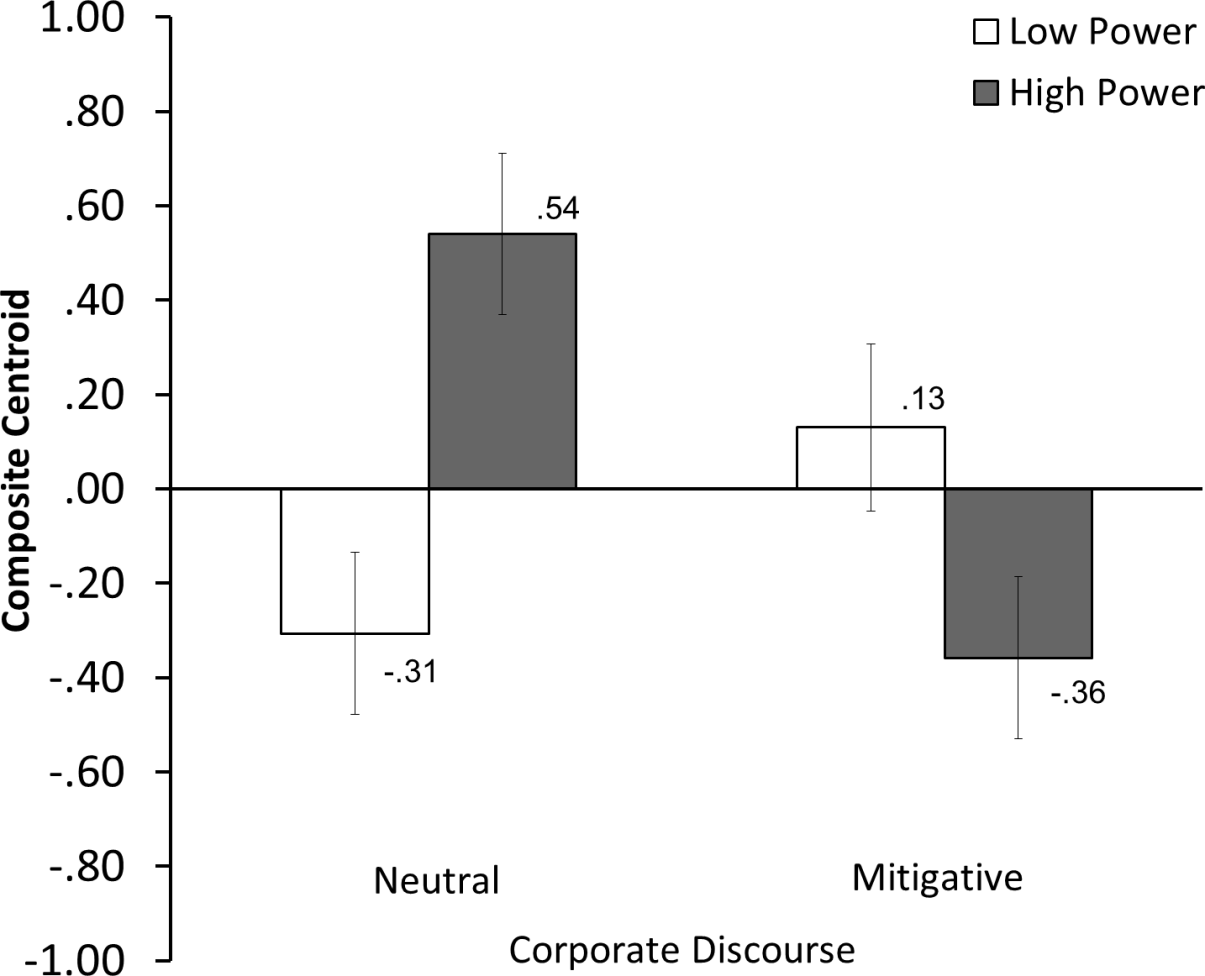
Group centroid means for the power ✕ discourse interaction composite dependent variable (Study 2). Higher score indicates greater negative attitude toward the company. Error bars indicate standard errors.

With the multivariate composite as the dependent variable, we conducted a 2 (power) ✕ 2 (discourse) univariate ANOVA. See Fig 1 for the graphical representation of the group centroid means in each condition. Under neutral discourse, participants in the high power condition expressed significantly more negative attitudes toward the company compared to those in the low power condition (*t* (130) = -3.49, *p* < .001, *d* = -1.14); in contrast, under a mitigative discourse, those in the high power condition responded less negatively than those in the low power condition (*t* (130) = 1.98, *p* = .0496, *d* = 0.49). An alternate mean comparison indicated that the type of discourse affected attitudes especially when people were in a powerful position (*t* (130) = 3.70, *p* < .001, *d* = 0.89), while the effect was substantially weak when people were in a state of low power (*t* (130) = -1.77, *p* = .079, *d* = -0.59).

The results of the MANOVA supported our hypotheses. Those who were accredited power over a corporate predicament tended to shift their attitudes in greater accordance with the type of discourse offered by the company. In an absence of a mitigative discourse, the high-power individuals responded with stronger negativity toward the accused company than did the low-power individuals. On the other hand, in the presence of a mitigative discourse, the high-power individuals responded with less negativity than the low-power individuals.

## General discussion

We examined through two studies whether people’s experiences of power influence their responses to corporate transgressions. We approached the variable of power using two types of methodologies: experimental priming of power (Study 1) and inducing one’s sense of direct influence over the actions of the accused company (Study 2). Based on the Situated Focus Theory of power [20], we presumed that people who experience a possession of power would respond more in line with the type of discourse offered by the firm—that is, whether it includes mitigative elements (e.g., apology)—than those feeling less powerful at the moment.

We obtained general support for our hypotheses. First, in the face of a neutral discourse, the high-power individuals responded with stronger negativity aimed at the company than did the low-power individuals. They expressed greater reluctance to use the company’s products, along with stronger retributive intentions and negative affect. According to the Situated Focus Theory, such results are explained in terms of power enabling people to focus on the activated goal (e.g., to punish the company) and executing goal-consistent responses.

Second, however, such tendencies did not persist when the company admitted its responsibility and publicly apologized. Instead of retaining the unfavorable attitudes aimed at the company, the high-power individuals altered their attitudes in response to the mitigative discourse. In Study 1, they were no more reluctant to use the company’s products than the low-power individuals; in Study 2, they were more willing to use the products and expressed less negative views and intentions toward the company than their low-power counterparts. Our data demonstrate that power does not simply make people more punitive or more benevolent in a uniform manner. Rather, the results signify the core element of the Situated Focus Theory—that power enhances people’s coordination to situationally-salient goals or expectancies and contributes to greater behavioral flexibility [19].

Centering on the Situated Focus Theory, we aimed to integrate the theoretical model of power into the research on consumer-corporate relationship. Whereas existing research have considered the effect of power on daily consumption behaviors [10], our data demonstrate that power potentially holds considerable impact on how consumers respond in cases of corporate transgressions. We argue that the empirical focus on power in this domain is especially important since the inherent power relationship between consumers and corporate firms is amidst a rapid change. That is, taking advantage of the Internet, consumers can now communicate with other like-minded consumers, gain increased consumer exit options from the market, and possibly influence companies and their brands [11, 17, 18]. Our data suggest, on one hand, that consumer empowerment drives consumers to show negative psychological (and potentially behavioral) reactions to misbehaving firms. On the other hand, our results also suggest that empowerment lead consumers to respond more positively if the firm is able to give out a valid apology. In overall, our findings portray that as a result of consumer empowerment, the demeanor of companies after their misconduct may take on even greater importance in consumer-corporate relationships. Future studies can develop on this point by testing the relevance of people’s Internet usage relative to the present research.

We may also place the current research within the literature on conflict resolution. There is an accumulation of findings focusing on apology’s effects toward forgiveness [54, 55], with the emphasis on the role of apologies extending to more applied areas, such as in the legal [56] or the political domain [57]. Present research contributes to such line of research in that we considered apology given publicly, conveyed through corporate discourses. Existing study reports that public apologies by organizational entities generally fail to promote forgiveness [32]. In our study, such ineffectiveness of a corporate discourse appeared among those in the low-power condition. Given that empowerment fostered forgiveness among our participants, the data suggest that power is perhaps a necessary precondition for forgiveness when apologies are administered publicly rather than privately.

By experimentally inducing feelings of power, we demonstrated a causal link between people’s subjective experiences of power and the attitudes they express toward corporate transgressions. Meanwhile, the approach is limited by the fact that we have only focused on artificial forms of power. Applying other methodologies (e.g., general surveys), future studies can test the model based on the forms of power consumers mundanely experience.

In addition, to broaden the scope of research, studies can capture types of consumer–corporate relationships that we did not address in the current study. In our experiments, people received information about corporate transgressions in the form of media coverages, thus generating attitudes as an uninvolved third-party. When directly involved, people may be more motivated to sanction the company and take direct actions; therefore, the feelings of being powerful or powerless may be more salient.

Despite the limitations, our findings provide a framework to systematically understand how power characterizes various forms of relationships a consumer can have with a company.

## Supporting information

S1 Appendix Vignettes used in the studies. (PDF)

S1 Table Factor analysis result for Study 2. (PDF)

S1 Dataset Dataset of Study 1. (CSV)

S2 Dataset Dataset of Study 2. (CSV)
