# Supplementary material for: Impact of consumer power on consumers’ reactions to corporate transgression
Source: PLoS One. 2018 May 3;13(5):e0196819. doi: 10.1371/journal.pone.0196819 (PMC5933779; doi:10.1371/journal.pone.0196819)
Supplement: S1 Appendix — (PDF) [file pone.0196819.s001.pdf]

S1 Appendix

Vignettes used in Study 1

Corporate transgression

On November 13th, 2005, a sudden fire broke out on a cargo truck while it was driving in the Yamagata prefecture, wounding its 36-year-old male driver. The type of the vehicle was a 2004 “Y-FV” manufactured by Y Motors, Inc. After the accident, several similar cases were reported with the same type of vehicle in other regions. (Newspaper excerpt)

Corporate discourse

Numerous media outlets have speculated about the cause of the accident, debating whether the fault was on the driver’s side or the vehicle’s deficiency. To bring the heated debate under control, a press conference was held by Y Motors. At the conference, managers of Y Motors expressed the following.

Mitigative discourse

“We have completed our investigation into the cause of the recently occurred vehicle fire incident involving our product, Y-FV. Consequently, we discovered that pressing down on the gas and the brake pedals generated a certain amount of heat. Such heat generation is not problematic if you use our company’s genuine mat product; however, the victims of the accident had been using a commercially available, unauthorized product, and we have identified this as the cause of the fire. Although we had been making announcements on the potential risk of not using a genuine product, we admit that we inadequately disseminated this information. We also feel that we insufficiently addressed the heat generation issue. We deeply apologize to the victim of the accident.”

Neutral discourse

“Regarding the recently occurred vehicle fire incident involving our product, Y-FV, we are currently investigating and we plan to provide information as soon as we determine the cause. There have been media reports suggesting deficiency in our product as the cause of the accident; however, we cannot say anything at this point until the

investigation is complete. We ask you to look forward to a further announcement that we will arrange as soon as we identify the cause of the problem."

Vignettes used in Study 2

Corporate transgression

On September 26th, the Osaka prefectural police department began their investigation on what is potentially a case of professional negligence: a 20-year-old female university student living in Seto city, Aichi prefecture, suffered severe burns on her head and face when her newly purchased hair dryer, a product of Breton Electronics (located in Suita-city, Osaka), suddenly blew out fire.

The dryer had been purchased just 2 weeks prior the incident, and reports show that there was nothing particularly wrong about how the student was using the product. According to the local officials, the accident occurred because the overheat prevention fan inside the product stopped operating due to a loose connection in its power circuit.

Through interviews with individuals within Breton Electronics, the police have found questionable details regarding the company's manufacturing and stock management processes of the product. In addition, it has been reported that strong impact may have been given to the product during its shipping process. Based on such interview reports, the local officials are investigating the specific cause of the product's malfunction.

Mitigative discourse

"We deeply regret the incident where an individual incurred a severe injury due to our product. It has become evident that there had been an inappropriate handling of the product during its shipping process, and we feel deeply responsible for not fully keeping track and taking control of the transportation conditions. Furthermore, we acknowledge that the inadequacy in our company's manufacturing and management of the product was also one of the causes of the accident. We will make whatever effort is necessary to prevent a recurrence of the accident. We deeply apologize for what happened."

Neutral discourse

"Regarding the incident that involved our product, we are currently investigating, and we plan to provide information as soon as we determine the cause. There have been media reports suggesting inadequacy in our company's manufacturing and management

of the product or an inappropriate handling of the product during the shipment process as the cause of the accident; however, we cannot say anything at this point until the investigation is complete. We ask you to look forward to a further announcement that we will arrange as soon as we identify the cause of the problem."
