# Supplementary material for: Impact of consumer power on consumers’ reactions to corporate transgression
Source: PLoS One. 2018 May 3;13(5):e0196819. doi: 10.1371/journal.pone.0196819 (PMC5933779; doi:10.1371/journal.pone.0196819)
Supplement: S1 Table — (PDF) [file pone.0196819.s002.pdf]

# S1 Table

# Factorial structure of attitude measures in Study 2

*Promax-rotated factor loadings, communalities, and Cronbach's alphas for the dependent measures of Study 2.*

| | Factor loadings | | | |
|---|---|---|---|---|
| | 1 | 2 | 3 | Communality |
| Antipathy toward Breton Electronics | **.93** | - .02 | - .05 | .79 |
| Anger toward Breton Electronics | **.91** | - .06 | - .08 | .71 |
| Distaste toward Breton Electronics | **.77** | .05 | .12 | .76 |
| As a consumer, I forgive Breton Electronics | - .09 | **- .86** | .15 | .70 |
| I am willing to use Breton Electronics' products if there is such chance | - .07 | **- .82** | .00 | .63 |
| I feel trust in Breton Electronics | .08 | **- .71** | - .05 | .49 |
| I prefer not to use Breton Electronics' products | .06 | **.48** | .20 | .39 |
| Breton Electronics should be given legal sanctions | .01 | - .01 | **.83** | .69 |
| I want Breton Electronics to take responsibility | - .09 | - .03 | **.68** | .38 |
| Breton Electronics should be given societal sanctions | .27 | .06 | **.44** | .44 |

*Note*. Boldface indicates highest factor loadings. Factor 1 = Negative affect; Factor 2 = General approach/avoidance to company; Factor 3 = Retributive intention.
